# The problem of selection bias in studies of pre-mRNA splicing

**DOI:** 10.1038/s41467-023-37650-2

**Published:** 2023-04-08

**Authors:** Zachary W. Dwyer, Jeffrey A. Pleiss

**Affiliations:** Department of Molecular Biology and Genetics, Cornell University, Ithaca, New York USA

**Keywords:** Alternative splicing, RNA splicing

## Abstract

In this comment, the authors discuss the potentially widespread problem of selection bias in drawing biological conclusions from RNA sequencing data.

## Teaser

Here we demonstrate how selection bias in studies of pre-mRNA splicing can inadvertently lead to inaccurate biological conclusions which nevertheless appear statistically robust. We argue that this may be a pervasive problem with potentially significant consequences for the field.

## The problem

Selection bias, also sometimes referred to as sample bias or sample selection bias, generally refers to a distortion (bias) of statistical testing that results from the way that samples are collected (selection). While this problem has been well understood in clinical and social sciences for quite some time^1–3^, its significance in molecular biology is less widely appreciated. Work from Oshlack’s group^4,5^ provides perhaps the most compelling demonstration of the issue of selection bias in molecular biology, wherein they demonstrate its impact on RNA-seq experiments where analyses of differential gene expression are coupled to analyses of GO-term enrichment. Oshlack’s group highlights a major pitfall in studies like this which is that not all transcripts are measured with the same statistical power in a typical RNA-seq experiment: because longer and more highly expressed transcripts are sampled more frequently than are shorter and lower expressed transcripts, there is more statistical power to identify long and/or highly expressed transcripts as being differentially expressed. The result of this is a distortion in the statistics that measure enrichment: the set of transcripts identified as differentially expressed is dependent not only on their biological behavior but on distinct properties that enhance their capacity for detection in the experiment. Importantly, because splicing-informative reads are rare within standard RNA-seq datasets^6^, the problem of selection bias can be particularly problematic in studies involving pre-mRNA splicing. We therefore aim to raise awareness of the problem of selection bias in analyses of next-generation sequencing studies designed to understand quantitative changes in pre-mRNA splicing.

## Causes and consequences of selection bias

To demonstrate both the problem of selection bias and the deleterious consequences of this bias in studies of pre-mRNA splicing, we designed a simple experiment that examines changes in splicing in the background of a well-characterized genetic variant of a canonical spliceosomal component: the RNA helicase Prp2. Work from several groups has established a role for Prp2 in rearranging the spliceosome prior to the first catalytic step^7–10^, and as such a reasonable expectation is that loss of Prp2 function would result in defective splicing for all (or nearly all) expressed transcripts. Using a targeted sequencing approach termed Multiplexed Primer Extension Sequencing, or MPE-seq^6,11^, which massively enriches for splicing informative reads, we generated rich datasets, equivalent to ~ an entire lane of NextSeq550 sequencing for each of triplicate samples from a budding yeast strain harboring the conditional *prp2-1* allele and a matched wild-type strain. To demonstrate the effect of sequencing depth on experimental outcome, we then computationally downsampled this large experiment to generate three smaller subsets of data, equivalent in an RNA-seq setting to what could be considered high, medium, and low sized experiments, or ~ 80, 40, and 20 million reads per replicate, respectively.

**Fig. 1:**
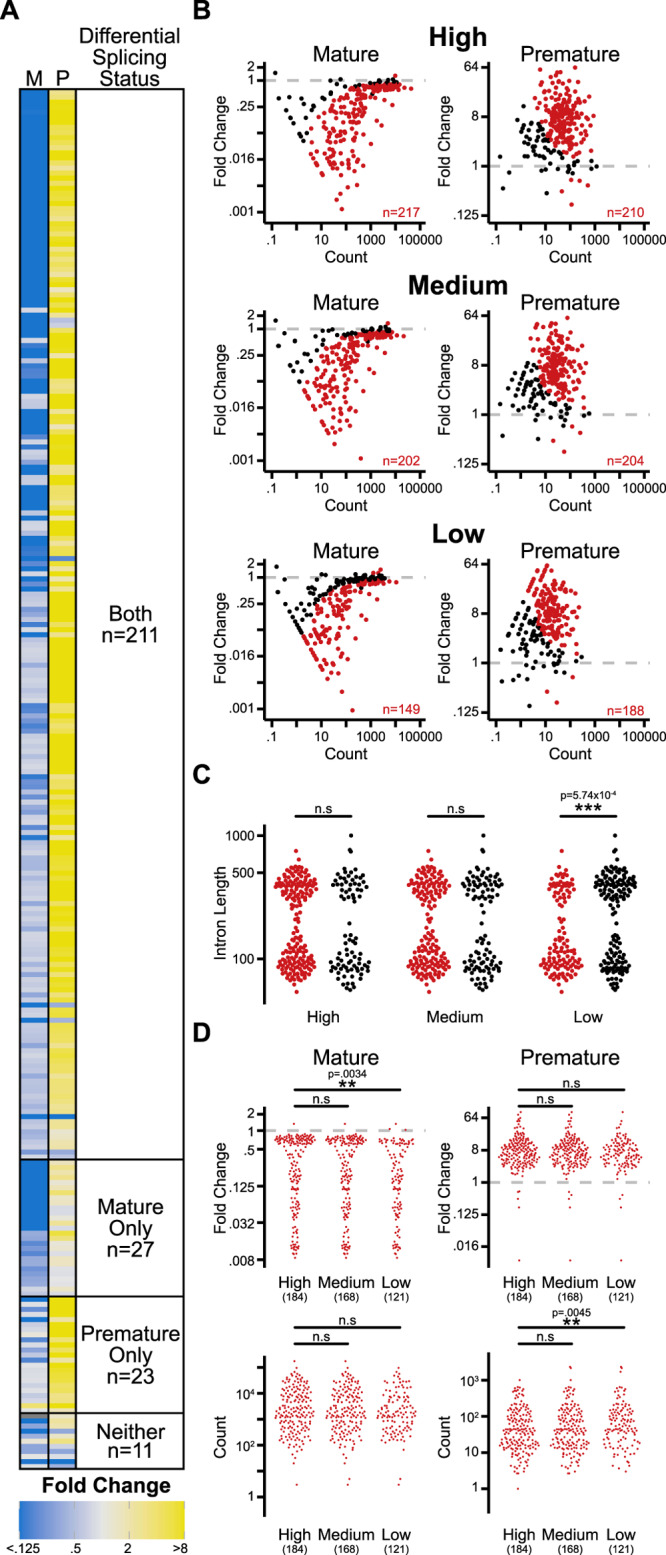
Selection bias introduces false correlations at insufficient read depth. Comparison of genome-wide splicing status in a *prp2-1* harboring strain relative to a matched wild-type strain after a ten min. shift to the non-permissive temperature (37 °C). **A** Heat map of fold change broken into categories of introns where both mature and premature, only mature, only premature, and neither mature nor premature supporting reads are significantly different between the Prp2 mutant and wild type as measured by DESeq2 at a multiple hypothesis corrected value of 0.05. **B** Fold change in number of mature or premature supporting reads as a function of expression between Prp2 mutant and wild type after downsampling to library sizes of 800,000 (High) MPE-seq reads to 400,000 (Medium) and 200,000 (Low) reads as measured by DESeq2. Red points are statistically significantly different at a multiple hypothesis corrected value of 0.05. **C** Length of introns that have (red) or do not have (black) significant difference in both premature and mature supporting reads as a function of read depth. One-sided Mann–Whitney test was performed to determine significance. **D** Fold change (upper) and count total (lower) for mature and premature supporting reads of introns that are significantly different in both mature and premature supporting counts at each downsampling. Two-sided Mann–Whitney test was performed to determine significance.

To analyze these datasets, for each intron-containing gene in the genome we calculated the fold change in abundance of reads corresponding to both premature and mature isoforms and then assessed these for differential expression using DESeq2 (Ref. ^12^). Of the 272 splicing events profiled in the full dataset, 261 demonstrated statistically significant differential splicing in the mutant relative to wildtype (Fig. 1A). For the majority of these (211), both the premature and mature versions of the transcript were detected as differentially expressed, whereas a smaller number of transcripts displayed differential expression of only one of the two isoforms; presumably reflecting different intrinsic properties of the rates of synthesis or degradation of these transcripts^13^. Importantly, the absence of evidence for differential expression of either splicing isoform for the remaining 11 transcripts in this experiment cannot be interpreted as evidence of an absence of an impact of the *prp2-1* variant on these transcripts. While such a biological conclusion might be true, it could also be that the design of this experiment was flawed—from a biological standpoint—perhaps because these transcripts were not actively expressed under the chosen conditions. Equally plausible, however, is the possibility that the experiment was technically flawed because even at this high sequencing depth it lacked sufficient statistical power to detect real changes in the splicing efficiency of these transcripts.

The consequences of decreased statistical power become readily apparent when considering our analyses of the downsampled datasets (Fig. 1B) wherein ever decreasing numbers of events were detected as differentially expressed with statistical significance as the read depth decreased. Whereas analysis of the full dataset demonstrated with statistical significance the widespread impact of *prp2-1* on the genome-wide splicing outcome, in standard sized experiments many of these splicing events lack the statistical power to be ‘selected’ within the class of transcripts considered impacted by *prp2-1*: the selection bias problem. Importantly, as Oshlak’s group previously demonstrated for standard gene expression studies, loss of statistical power does not occur evenly across the complement of genome-wide events being monitored, but rather occurs as a function of intrinsic properties of those targets which may or may not be important in the context of the biological problem being examined. For example, Fig. 1C shows a comparison of the lengths of the introns that were identified as impacted or not at each of the different experimental depths. Whereas no length difference was apparent between these classes at the High and Middle sizes, in the Low dataset a strong and statistically significant difference in the intron lengths was observed between the classes. While this result might suggest that short introns are more sensitized to loss of Prp2 function, the underlying data are more consistent with this being a biologically meaningless result of the loss of statistical power. As with most approaches for statistical testing, DESeq2 considers two important properties of the data in determining significance: the effect size, or difference in expression between the experimental and control samples; and the variance associated with the underlying measurements. For the relatively highly sampled mature isoforms (Fig. 1d, left), the subset of introns identified as differentially expressed are not characterized by higher read counts, but rather by larger fold-changes. Small fold-changes in the expression of the mature mRNA only surpass the significance threshold in the highly sampled dataset where overall variance is decreased. By contrast, for the relatively rare premature isoforms, the subset identified as differentially expressed is biased towards those that are highly sampled: even large fold-changes in differential expression fail to be deemed statistically significant if read depth is low (where variance is naturally higher).

## Practical implications of selection bias

**Fig. 2:**
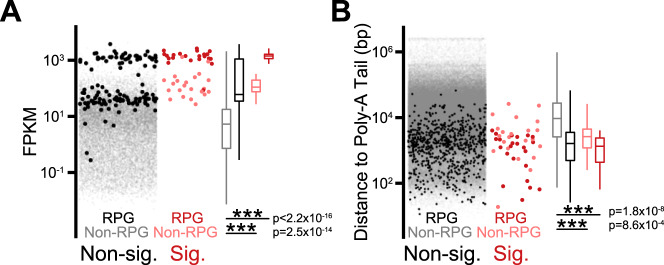
Selection bias in identified intron retention events. **A** Expression (measured in Fragments per Kilobase per Million Reads) of all introns versus those identified as retained broken out by ribosomal protein genes (RPG) and non-ribosomal protein genes (Non-RPG). **B** Distance from intron to poly-A Tail for all introns versus those identified as retained, broken out by RPG and Non-RPG. Two-sided Mann–Whitney test was performed to determine significance. For all boxplots, the median value is represented by the center line, box limits represent the 25th and 75th quartiles, and whiskers are 1.5x the interquartile range.

The above data demonstrate how selection bias has the potential to impact splicing studies, and we argue that this problem is likely pervasive in the field. To illustrate this, we consider here the data from one study which examined the role of the splicing factor HTATSF1, the ortholog of yeast Cus2^14^. As a core component of the U2 snRNP, and building off of significant prior work demonstrating a role for Cus2 in stabilizing a core structure of the U2 snRNA^15–17^, a reasonable expectation is that loss of HTATSF1/Cus2 activity would lead to decreased splicing efficiency across the complement of genome-wide substrates, akin to our expectations and observations for loss of Prp2 function as presented above (Fig. 1). In contrast, based on a knock-down experiment in mouse embryonic stem cells, it was reported that HTATSF1 appeared to function as a regulator of intron retention specifically in ribosomal proteins. While compelling statistical support was provided for intron retention within 45 different transcripts (many of which are involved in ribosome biogenesis and assembly), we wondered whether these transcripts were indeed uniquely impacted by loss of HTATSF1, or whether these were among the subset of transcripts for which there was sufficient statistical power in the experiment to detect a change in splicing efficiency. We therefore asked if the underlying data suggested a bias in the subset of identified events by examining two parameters which are expected to influence pre-mRNA detection: expression level of the host transcript, and distance between the end of the affected intron and the polyadenylation site for that transcript. Our motivation for examining this second feature is that most RNA-seq protocols, including the one employed in Corsini et al.^14^, utilize a poly(A)^+^ enrichment step. Because splicing is coupled to transcription^18^, the likelihood of a retained intron being detected within a poly(A)^+^ pool of RNA is expected to be highest for those introns located closest to the polyadenylation site, as these would have the least amount of time for removal prior to polyadenylation. As shown in Fig. 2, the events identified in Corsini et al.^14^ appear biased for each of these properties such that they would be expected to have much greater statistical power for detecting differential expression than most of the other events in the genome. As such, while we do not question the role of coordinated control of ribosome biogenesis, our analysis suggests that the exact extent to which HTATSF1 controls splicing and intron retention specifically in ribosomal proteins could have been impacted by selection bias: an enhanced capacity to detect changes in splicing of these transcripts rather than a specific defect in their processing.

## How can selection bias be mitigated?

Here we argue that selection bias not only can have deleterious impacts on RNA-seq-based studies of pre-mRNA splicing, but in fact has had and will continue to have such impacts unless and until knowledge of this problem and its potential solutions becomes widely appreciated. While this problem has been previously noted^19^, our hope here is to bring renewed attention to this issue. Importantly, while this problem has been carefully considered in the context of standard gene expression studies^4,5^, a cursory examination of the literature suggests this problem nevertheless continues to pervade current work. Whereas Oshlack provided an elegant mathematical approach for mitigating the impacts of selection bias in GO-term enrichment analyses, we unfortunately offer no such solution here. While we hope that others might provide tools to mitigate this problem in the future, we suggest that the simplest solution to mitigate this problem is the use of approaches that either enrich for, or otherwise increase the number of, splicing-informative reads across the complement of genomic substrates^11,20^, thereby reducing the differences in sampling across the datasets.

Importantly, while the work examined here involved short-read, Illumina-based sequencing, we note that this is not a problem unique to this platform but rather reflects a fundamental statistical challenge associated with analyzing datasets with small numbers of measured events. As such, we expect that this problem will be even greater in analyses of datasets from long-read sequencing platforms where the number of reads per experiment is typically much lower, and likewise in single-cell experiments where the number of reads per cell is dramatically reduced. Indeed, evidence of such bias in single-cell experiments has been recently demonstrated^21^. Similarly, while many software packages have been developed which enable more sensitive detection of splicing ‘hits’ within an experiment^22–26^, the problem of selection bias is fundamentally an issue of how to handle those events which are not identified as hits: those wherein the absence of evidence cannot be interpreted as evidence of absence. We are unaware of any software packages that are widely available today that account for this problem but hope that such solutions will soon arise. In the meantime, we conclude by noting that as users of these technologies, whether that be as experimentalists generating and analyzing such data, or as consumers evaluating the work of others, it will be essential for all of us to consider the possibility that an apparently statistically significant conclusion may not reflect a meaningful biological property but instead may be the result of a statistical aberration.

## Methods

### Cell growth

Wild-type cells and those harboring the *prp2-1* allele were streaked from glycerol stocks onto solid rich media (YPD) and grown at the permissive temperature (25 °C) for three days. In triplicate, single colonies were inoculated into 5 mL of YPD and grown at 25 °C with shaking at 200 rpm overnight. Cultures were back-diluted into 20 mL of YPD to an OD_600_ of 0.05 and incubated at 25 °C with shaking at 200 rpm. Upon reaching an OD_600_ of approximately 0.75, cultures were transferred to a 37 °C shaking water bath (200 rpm) for 10 min. Cells were collected via vacuum filtration and pellets were immediately flash-frozen in liquid nitrogen and stored at −80 °C.

### MPE-seq library preparation

RNA was purified and MPE-seq libraries were prepared as previously described^6^ with the exception that biotin-11-dUTP was used in place of aminoallyl-dUTP during reverse transcription such that no separate biotin coupling step was necessary. Instead, following hydroxide treatment an elution volume of 50 µL was used during the zymo column clean-up which went immediately into the first bead purification.

### Downsampling

Using a custom script, each read was assigned a random number from 0 to 1 and sorted based on their random number. The top 800,000, 400,000, and 200,000 were included in the high, medium, and low datasets, respectively. Random number generation used a seed value of 1 to allow future reproducibility.

### Alignment and quantification

The full and downsampled datasets were processed as follows: Reads were trimmed of sequencing adapters using fastp^27^ with the following parameters: --adapter_sequence CTGTCTCTTATACACATCT --adapter_sequence_r2 CTGTCTCTTATACACATCT. Trimmed reads were aligned to the R64-2-1 genome release from SGD with hisat2^28^ with the following parameters: --max-intronlen 2000 --no-unal and reads with MAPQ scores below 5 were removed with samtools^29^. Unspliced and spliced counts were obtained with a custom script based on HTSeq-count^30^. DESeq2^12^ was used to assess differential splicing.

### Data processing

FPKM values for host genes and identified retained introns were obtained from Corsini et al. (GEO GSM2535498 and Table S2 therein, respectively)^14^. All mm9 UCSC introns were broken into groups based on whether they were identified as significant and whether they are ribosomal protein genes. Distances from the end of each distinct intron (as determined by their chromosome, start, and stop positions) and the end of the host transcript were calculated from genomic coordinates. In the case that an intron existed within multiple transcript isoforms, the shortest isoform was considered.

### Reporting summary

Further information on research design is available in the Nature Portfolio Reporting Summary linked to this article.

## Supplementary information

Reporting Summary

## Data Availability

All newly generated sequencing data are available through NCBI’s Gene Expression Omnibus (GEO) at accession number GSE160046, and the data previously reported^14^ obtained under accession number GSM2535498.

## Code availability

Code for basic analysis steps is available on GitHub (https://github.com/zdwyer/Problem-of-Selection-Bias).
